# Hypersensitivity Reactions to Monoclonal Antibodies in Children

**DOI:** 10.3390/medicina56050232

**Published:** 2020-05-12

**Authors:** Francesca Mori, Francesca Saretta, Annamaria Bianchi, Giuseppe Crisafulli, Silvia Caimmi, Lucia Liotti, Paolo Bottau, Fabrizio Franceschini, Claudia Paglialunga, Giampaolo Ricci, Angelica Santoro, Carlo Caffarelli

**Affiliations:** 1 Allergy Unit, Meyer Children’s Hospital, 50139 Florence, Italy; f.mori@meyer.it; 2 SC Pediatria, Ospedale Latisana-Palmanova, Dipartimento Materno-Infantile Azienda Sanitaria Universitaria Friuli Centrale, 33057 Palmanova (UD), Italy; francescasaretta@gmail.com; 3 Pediatria, Ospedale San Camillo, 00149 Roma, Italy; annamaria.bianchi9@yahoo.it; 4 UO Allergologia, Dipartimento di Pediatria, Università di Messina, 98124 Messina, Italy; crisafullig@unime.it; 5 Clinica Pediatrica Policlinico San Matteo, University di Pavia, 27100 Pavia, Italy; sissi_del_78@hotmail.com; 6 Pediatria, Ospedale Principi di Piemonte, 60019 Senigallia, Italy; lucialiotti@libero.it; 7 Pediatria e Neonatologia, Ospedale di Imola, 40026 Imola, Italy; paolo.bottau@gmail.com; 8 UOC Pediatria, Azienda Ospedaliero-Universitaria “Ospedali Riuniti”, 60020 Ancona, Italy; allped@libero.it; 9 UOC di Pediatria, Azienda Ospedaliera-Universitaria “Consorziale-Policlinico”, Ospedale Pediatrico Giovanni XXIII, 70123 Bari, Italy; clapag07@gmail.com; 10 Pediatric Unit, Department of Medical and Surgical Sciences, University of Bologna, 40138 Bologna, Italy; giampaolo.ricci@unibo.it; 11 Clinica Pediatrica, Dipartimento Medicina e Chirurgia, Università di Parma, 43126 Parma, Italy; angelica.santoro204@gmail.com

**Keywords:** monoclonal antibodies, biologic drug, drug allergy, hypersensitivity reactions, challenge, desensitization, prick test

## Abstract

Biologic drugs are widely used in pediatric medicine. Monoclonal antibodies (mAbs) in particular are a therapeutic option for rheumatic, autoinflammatory and oncologic diseases. Adverse drug reactions and hypersensitivity reactions (HSR) to mAbs may occur in children. Clinical presentation of HSRs to mAbs can be classified according to phenotypes in infusion-related reactions, cytokine release syndrome, both alpha type reactions and type I (IgE/non-IgE), type III, and type IV reactions, all beta-type reactions. The aim of this review is to focus on HSRs associated with the most frequent mAbs in childhood, with particular attention to beta-type reactions. When a reaction to mAbs is suspected a diagnostic work-up including in-vivo and in-vitro testing should be performed. A drug provocation test is recommended only when no alternative drugs are available. In selected patients with immediate IgE-mediated drug allergy a desensitization protocol is indicated. Despite the heavy use of mAbs in childhood, studies evaluating the reliability of diagnostic test are lacking. Although desensitization may be effective in reducing the risk of reactions in children, standardized pediatric protocols are still not available.

## 1. Introduction

As stated by the World Health Organization (WHO) a “biologic” drug (BD) is a “biotherapeutic protein product prepared by recombinant DNA technology” [1]. Among BD there are vaccines, hormones, blood derivates, growth factors, immunoglobulins and monoclonal antibodies (mAbs). mAbs will be the focus of this review. From 1985 about a hundred different mAbs drugs have been discovered. In 1995 the International Nonproprietary Name expert group of the WHO decided the naming rules for mAb drugs [2]. A mAb name is composed of: a prefix (specific and unique drug name); two subitems which describe the target (e.g., “tu for tumor”, “so” for bone) and the source from which the mAb is derived (“u” for human, “o” for mouse, “xi” chimeric (with 25% of the murine fraction in the fragment antigen binding (Fab), “zu” humanized (with 2–5% of the murine fraction in the Fab fragment); a suffix which is for all mAbs “mab”. Many mAbs are not specifically approved for pediatric use. The most frequently used BDs in the pediatric age group are listed as follows. Etanercept, adalimumab, abatacept, and tocilizumab are used for juvenile idiopathic arthritis (JIA) and other rheumatic diseases. Anakinra and canakinumab are often used for cryopyrin-associated periodic syndrome (CAPS) and other auto-inflammatory syndromes (e.g., familial Mediterranean fever (FMF) and mevalonate kinase deficiency (MKD)). Rituximab is used for idiopathic thrombocytopenic purpura (ITP), while omalizumab, benralizumab, mepolizumab are used for severe eosinophilic asthma. Omalizumab is used for chronic urticaria, dupilumab for atopic dermatitis (AD), infliximab for bowel inflammatory diseases (BID). Characteristics of the mAbs currently approved by Food and Drug Administration (FDA) and European Medicines Agency (EMA) for pediatric use are listed in Table 1.

BDs are proteins with a high molecular weight and some are partially of non-human origin. As for all medications, mAb can lead to adverse drug reactions (ADR). ADRs to mAbs differ from those elicited by other drugs. mAbs act as autologous proteins and not as chemical compounds; they are metabolized as proteins and not as chemical molecules. Furthermore, these reactions are often due to immune activity in response to themselves [32]. Consequently, different types of ADR to mAbs were classified according to Pichler and to the mechanism of action [32]. They are as follows: (1) type alpha, high cytokine and cytokine release syndrome (CRS); (2) type beta (hypersensitivity) can be IgE mediated, IgG mediated or T cell mediated (Coombs’ and Gell’s classification); (3) type gamma (immune (cytokine) imbalance syndromes) is due to the intrinsic activity of drugs leading to infections or malignancy because of immunosuppression, or immune imbalance (e.g., reactivation of tuberculosis induced by anti-TNF-alpha mAb); (4) type delta (cross-reactivity) is due to the action of the drug on molecules overexpressed in tumor cells (e.g., acneiform rash induced by cetuximab); (5) type epsilon (nonimmunological side-effects), in which the immune system is not involved. More recently, the phenotype and endotype of hypersensitivity reaction (HSR) to mAbs have been described [33]. They consist of infusion-related reaction (IRR), CRS, type I (IgE/non-IgE), type III, and type IV reactions. This review will focus on HSRs to mAbs in childhood, particularly on beta-type reactions.

## 2. HSR to mAbs

A number of studies have reported safety data on mAbs. However, most trials in children are small case series. Moreover, in many studies HSRs are incorporated in ADR. HSRs are therefore missed [34]. For the purpose of this review, only data from approved and most-frequently-used mAbs in childhood are presented (Table 1). HSRs to mAbs are less common for fully human or humanized mAbs that elicit lower immunogenic responses than mAbs with lower degree of humanization [35]. They also depend on the involved mechanism (IgE-mediated, IgG-mediated etc.). Finally, reactions could be induced by potentially allergenic excipients including mannitol (adalimumab, basiliximab, palivizumab, etanercept), polysorbate (adalimumab, infliximab, omalizumab, canakinumab), latex (adalimumab, etanercept, anakinra), and trometamol (etanercept) [36].

### 2.1. Infliximab

Infliximab is a mAb which works as anti-TNF-α and it is currently approved for treatment of Crohn’s disease (CD) and in ulcerative colitis (UC) in children from 6 years of age. Immediate HSRs to infliximab appear in 10% of patients [13,14,15] while delayed HSRs (ranging from localized erythema to life-threatening reactions with blisters, erosions and bullae involving the mucus membranes) are less common [16], including different type of skin reactions [37]. In a large pediatric study [17], infliximab was well tolerated. IRR developed in 16.5% of patients (1652 doses in 243 children). No HSR was reported. In the study by Kugathasan et al. [38] on re-treatment with infliximab for CD, a lower rate of severe systemic reaction in children compared to adults (11 adults vs 1 child, *p* = 0.02), was observed. No delayed reaction was recorded in children. Ducharme et al. [14] analyzed a group of 3161 patients, age range 10-92 years, mean 44 years. Indications for treatment were CD and rheumatoid arthritis (RA). ADRs both immediate (from mild reactions to anaphylaxis) and delayed (i.e., rashes, flu-like symptoms, headache) were observed in 18.9% of patients. Most reactions were mild (50.2%) and in 39.9% of cases were delayed. In patients <18 years old, 16 ADRs (4.8%) were recorded. In this study, younger age seems to be a risk factor (*p* < 0.01) for adverse drug reactions. Regarding IRR, El-Matary et al. [18] showed a large case-series of 4120 infliximab rapid infusions in 453 children (13.8–17.8 years) for CD. Most patients (59%) were pretreated with anti-allergic drugs and 35.5% were already treated with immunosuppressive drugs. IRR occurred in 4.6% of patients, and only two (0.4%) patients had to discontinue the treatment. Premedication with antihistamines was associated with fewer reactions (*p* = 0.002). In adults, antibodies against mAb reduces the efficacy of treatment and increases the risk of HSR, especially for infliximab [39,40,41,42]. A meta-analysis [43] on the immunogenicity of mAbs used for JIA showed that all mAbs induced antibodies: abatacept in 2.3–23.3% of cases, adalimumab 10.9–37%, anakinra 81.8%, canakinumab 3.1%, etanercept 0–21.9%, golimumab 46.8%, infliximab 36.6%, tocilizumab 0.5–7.5%, with a total pooled prevalence of 16.9%. In 4 of 20 patients treated with infliximab who had antibodies to infliximab, a possible anaphylactic reaction was observed, while none occurred in those who had lacked infliximab antibodies.

### 2.2. Adalimumab

Adalimumab is a mAb against TNF-alpha approved for JIA, plaque psoriasis, non-infectious uveitis and CD in pediatric age patients. Horneff et al. [5] recorded allergic reactions in 577 children treated with adalimumab for JIA, psoriasis and CD. Allergic reactions were observed in 41/274 (15%) children with JIA, in 7/111 (6.3%) psoriasis and in 19/192 (9.9%) CD. Similar results were observed by Faubion et al. [44] who enrolled 192 children in a phase 3 double-blind, placebo-controlled study to evaluate the usefulness and safety of adalimumab for CD. The study was designed to have a double-blind trial in the first step (IMAgINE 1) and a second step (IMAgINE 2) in which only the patients who have responded to the first step were entered. Allergic reactions (not otherwise specified) occurred at any time of the study at a rate of 14.9%. Other studies have evaluated the efficacy and safety of adalimumab in childhood for different diseases, but only adverse events were recorded with no specific mention of HSRs [45,46,47]. Marino et al., [6] analyzed the incidence of adalimumab anti-antibodies with a new method. In ten children treated with adalimumab for JIA, seven children had antibodies against adalimumab that seem to correlate with a lower efficacy of therapy.

### 2.3. Abatacept

Abatacept is a mAb composed by a fusion protein with the extracellular domain of CTLA-4 linked to a modified Fc portion of human IgG1. It modulates the CD80/CD86 complex and blocks the T cell activation signaling. It has been approved in children >6 years of age for moderate-to-severe JIA with unsatisfactory response to other therapy, including anti-TNF-alpha drugs. Between 2008 and 2018 the Paediatric Rheumatology International Trials Organization and Pediatric Rheumatology Collaborative Study Group have established the efficacy and safety of abatacept for the treatment of JIA [48,49,50] both intravenously and subcutaneously. An open-label multicenter study conducted in 20 Japanese children has recently reported no HSR or anaphylactic reactions [3]. In 1,843 subcutaneous abatacept, the incidence rate for HSRs to abatacept has been shown to be 2.4 per 10,000,000 person-day in adults [4].

### 2.4. Etanercept

Etanercept is a fusion protein mAb against TNF-alpha and works as soluble receptor with a high affinity for TNF-alpha. It is approved in children older than two years of age for the treatment of JIA with insufficient response to Methotrexate (MTX); in children older than 6 years of age for plaque psoriasis and in older children (>12 y/o) for PA and enthesitis-associated arthritis. Quismorio and colleagues [51] presented two cases of anaphylaxis to etanercept, along with other cases reported in the literature. All of them are referred to adults treated with etanercept for RA; that was the probable culprit drug. One patient was treated also with MTX [52,53,54]. No allergy tests (skin tests, provocation test) were performed to confirm the diagnosis. In a study by Puxeddu et al. [55] a group of 51 adults, who were treated with different anti-TNF-alpha drugs for rheumatic diseases, were evaluated for a possible HSR. Rates of HSR were 13.8% for infliximab, 5.3% for etanercept and 3.5% for adalimumab. In 3 of 8 patients with anaphylaxis to infliximab at first administration, intradermal tests (IDT) were performed with negative results, suggesting a non-IgE mediated reaction. In children, there were some reports of HSR to etanercept [10,11,12].

### 2.5. Tocilizumab

Tocilizumab is a mAb against IL-6 and it is approved for children older than 2 years of age for moderate-to-severe RA and systemic JIA with an unsatisfactory response to other disease-modifying antirheumatic drugs (DMARDs) and for CRS from chimeric antigen receptor T cell therapies (CAR-T therapy). Soyer et al. [30] investigated a group of 128 children (49.2% boys) with a mean age of 14.6 years (range 9.9–16.9 years) with different rheumatic diseases (mostly, 58%, with JIA) and treated with 32,494 doses of eight different mAbs. A local reaction at injection site (3 to anakinra, 1 to etanercept) occurred in 4/128 children. Six children had an immediate HSR. One child developed urticaria (canakinumab) and five children developed an anaphylactic reaction (three to tocilizumab and two to rituximab). None of them developed HSR at first injection. Anaphylactic reactions were moderate in three cases and severe in the other two cases. HSRs to mAbs had an incidence of 4.7% and anaphylaxis to mAbs of 3.9%. The frequency of HSR for infusion was 0.018%, whereas rate of severe HSR was 0.015%. Risk factors for HSR were exposure to multiple mAbs, more than 14 hospitalization/lifetime, active disease, and renal involvement. In a recent study [56], 413 children treated with different mAbs for rheumatic diseases were analyzed. Most of them had JIA 76.7% or 17.6% autoinflammatory disease (FMF, MKD, CAPS). 7.5% of JIA children had also FMF. The most frequent used drug was MTX (69.4%); among mAb, 31.4% of children received etanercept, 21.7% adalimumab, 17.1% anakinra, 15.4% canakinumab, 13.3% tocilizumab, 1.6% rituximab, 1.4% infliximab and 0.9% abatacept. In 4 out of 1722 infusions, one allergic reaction to infliximab and three to tocilizumab were recorded. In 41,113 subcutaneous injections, only one allergic reaction to MTX was observed. A child with severe systemic JIA developed angioedema due to anti-tocilizumab antibodies [30]. Rocchi et al. [57] have observed HSRs to tocilizumab in four (5.5%) out of 72 adults. All four patients had negative prick test but 3 of 4 had positive IDT. Tocilizumab has also been investigated in the off-label treatment of Takayasu arteritis in children and no ADR has been reported, while in other non-JIA rheumatic diseases some ADRs have been observed [58]. No HSR has been reported.

### 2.6. Rituximab

Rituximab is a mAb against the CD20 molecule on the B lymphocyte. It has been approved for treatment of B cells malignancies (lymphoma) and RA in adults. In children, it is used, off-label, for ITP [59], steroid-dependent nephrotic syndrome [60,61], and steroid-dependent Schonlein-Henoch purpura [62]. Most studies are small case-series, and none of them reported HSR to rituximab beside IRRs. A larger study by Dale et al. [26] has investigated the rate of ADRs with the off-label use of rituximab for central nervous disease in pediatric age patients in 144 children (age range 0.7–17 years). In 18 (12.5%) of 144 children infusion adverse events, including 3 (2%) cases of anaphylaxis, occurred. Anaphylaxis was treated with antihistamine and steroid, and no adrenaline was required. No difference was observed between patients who received antihistamine prophylaxis (13/106, 12%), and those who did not (5/38, 13%). In the children <5 years, there was no increased risk of infusion adverse events compared to children >5 years. Most of infusion adverse events were skin rash and fever. Several case series reported immediate HSRs to rituximab, mostly involving the adult population [27,28,29]. Most of immediate reactions occurred on first exposure, while delayed reactions were more common on episodic regimen [38]. There are several case reports of serum sickness reactions to rituximab [38,39,40,41,42,43,44,45,46,47,48,49,50,51,52,53,54,55,56,57,58,59,60,61,62,63], but none of them regarding children. IRRs seem to be more common in patients with lymphoma and less common in those pre-treated with steroids [64].

### 2.7. Omalizumab

Omalizumab is a humanized anti-IgE mAb for severe asthma in children from six years of age and for chronic urticaria from 12 years of age. Anaphylaxis to omalizumab is very rare and occurs in less than 0.2% of patients [21,22,23]. Chipps et al. [65] and Rodrigo et al. [66] found that anaphylaxis to omalizumab was rare. An incidence of 0.58% vs. placebo 1.04% (RR 0.51, *p* = 0.44) has been reported [58]. In the PROSE study [67] there were three anaphylaxes to omalizumab vs two for placebo and three for inhaled corticosteroids (ICS) boost. In the ITACA study [68] there was one anaphylactic reaction (mild) to final dose of omalizumab, and six to placebo. No anaphylactic episode related to omalizumab were observed in a French cohort [69,70], as well as in the study by Milgrom et al. [71]. Even in a case control study in patients with a history of omalizumab anaphylaxis [72], anaphylaxis was more frequent within the first three doses (39.3%) and within 1 h from administration (70% of cases), but no death or disability were recorded. Total number of doses, concomitant food allergy, female sex, and urticaria were identified as potential risk factor.

### 2.8. Mepolizumab

Mepolizumab is a mAb against IL-5 and it is approved in childhood for the treatment of severe eosinophilic asthma over 12 years of age for the FDA and over six years of age for EMA. Gupta et al. have studied [19] 36 children aged 6 to 11 years of age treated with mepolizumab, two study’s arms (40 mg or 100 mg). There was only one HSR in a child at 40 mg, characterized by mild itching. Since the reaction occurred less than 24 h after the first dose and lasted for 57 days, the study authors considered it as an HSR to mepolizumab. From the same clinical trial the authors also identified another allergic reaction in a child in the 100 mg arm [73]. In a group of 621 patients randomized to receive placebo (159) or different dosage of mepolizumab for severe eosinophilic asthma (75 mg or 250 mg or 750 mg) [20], the authors observed six patients with HSR possibly related to mepolizumab: three to placebo, one to 250 mg dosage and two to 750 mg dosage. No anaphylactic reactions were reported.

### 2.9. Dupilumab

Dupilumab is a mAb against IL-4/IL-13 for the treatment of severe AD in children >12 years of age, for the treatment of moderate-severe uncontrolled asthma with type 2 inflammation, and for severe AD. In a recent review on therapeutic options for severe asthma [74] dupilumab reduced asthma exacerbations and use of corticosteroids. As for safety, increase in eosinophilic count and more injection site reactions were reported in the dupilumab group. One case-series involving six eczematous children (two males, mean age 10.8 years) who received dupilumab, reported no ADR/HSR [75]. There are several on-going trials on the use of dupilumab in AD in childhood [7] and the safety issue is a main concern that should be further investigated.

### 2.10. Anakinra

Anakinra is a mAb to IL-1 used for the treatment of autoinflammatory diseases and for JIA in children older than 8 months of age and >10 kg of weight. Epcacac et al. [9] reported a severe anaphylactic reaction in a 6.5-year-old boy treated with anakinra for idiopathic recurrent pericarditis. Allergy tests have not been performed by this child, who was subsequently treated with canakinumab without HSRs. A similar case was reported in a two-year-old child treated with anakinra for JIA [76], while successful desensitization was performed in a seven-year-old child by Anton et al. [77]. Several similar cases have been reported in adults, followed by desensitization to anakinra [78,79,80,81,82,83].

### 2.11. Canakinumab

Canakinumab is another mAb against IL-1 and it is approved for children older than four years old for FDA and two years old for EMA. It is often used as an alternative to anakinra in case of ADR or HSR, and desensitization is not possible or feasible. Canakinumab seems to be a safe mAb; a recent study [9] in 14 children from a cohort of 714 patients followed for FMF did not reported any HSR.

### 2.12. Palivizumab

Palivizumab is a mAb against the A antigenic site of F protein of respiratory syncytial virus (RSV) and it is approved for newborns less than 35 weeks of age or 6 months of age or less at the beginning of season, or at high risk of complicated RSV disease (bronchopulmonary dysplasia (BPD), or congenital heart disease (CHD)). One case of anaphylaxis to palivizumab has been reported in a two-year-old girl [24]. The girl, affected by DiGeorge syndrome, tetralogy of Fallot, and prematurity lung disease, developed, at the second dose of the second year of treatment, an HSR characterized by vomiting, dyspnea, urticaria, angioedema, hypotension and tachycardia. She was promptly treated and palivizumab therapy discontinued. Allergy tests were not performed. One of the larger studies on safety of palivizumab [84] included only preterm babies and children aged <2 years with chronic lung disease. ADRs were documented in 40/565 children, none was severe. No HSR were reported. The CARESS study [85] analyzed children who have received at least one dose of palivizumab from 2008 to 2013, during RSV season, across 32 Canadian sites. The authors fulfilled this registry since the surveillance studies previously published included a limited number of patients. The Canadian registry included 13,025 infants: 63.1% aged 35 weeks or less, 11.1% aged <2 years with hemodynamically CHD, 7.5% with BDP, and 18.3% with other pre-existing, complex medical conditions at risk of complicated RSV infections. A total of 57,392 doses of palivizumab were administered. Six children presented a possible or probable adverse event, for a total of 14 events (0.05%). Provocation tests were positive in four children. The other two children were not challenged. The first child presented a generalized urticaria requiring hospitalization while the second angioedema. Children did not undergo allergy tests. Another recent study [25] also confirmed the safety of palivizumab in cystic fibrosis, reporting only one event in 92 patients.

## 3. Clinical Presentations of HSRs to mAbs

Common HSRs to mAb include the following phenotypes: IRR and CRS, both alpha-type reactions, type I (IgE/non IgE), type III, and type IV reactions (according to the classification of Gell and Coombs), and all beta-type reactions [33,34,35]. Delayed reactions have been reported mostly as serum-sickness-like reactions (SSLR), but also other severe cutaneous reactions have been observed (erythema multiforme (EM), drug reaction with eosinophilia and systemic symptoms (DRESS), acute generalized exanthematous pustulosis (AGEP), Steven-Johnson Syndrome (SYJS), toxic epidermal necrolysis (TEN) [36].

### 3.1. Infusion Related Reactions

IRR may be defined as: “any signs or symptoms experienced by patients during the infusion of pharmacologic or biologic agents or any event occurring on the first day of drug administration” [86]. Clinical expression may vary from mild to severe symptoms, including fever, chills/rigor, nausea, pain, headache, dyspnea, hypertension/hypotension. The mAbs release proinflammatory cytokines such as IL-6 and TNF-alpha from target cells [28]. The onset of the reaction occurs ten minutes to four hours after starting the administration or within 24 h from the first administration [87]. IRR usually develops on first administration [28]. In some of these IRRs an IgE-mechanism could be involved, and patients need to be carefully evaluated before continuing the treatment. Premedication with steroids/antihistamines and slower infusion rate could prevent the reaction. They are self-limiting on the following exposure [33].

### 3.2. Cytokines Release Syndrome

CRS is a systemic inflammatory reaction that can sometimes be life threatening. It is caused by large and rapid release of cytokines, such as interferon-gamma, TNF-alfa, IL-1 and IL-6, the latter identified as a possible biomarker of this type of reactions [33,88]. The cells releasing the responsible cytokines are not conclusively identified in all circumstances, but it is expected to involve CD8 + T cells, monocytes, natural killer cells and macrophages [89]. CRS quickly develops within minutes to a few hours following exposure and include specific symptoms (headache, low blood pressure, pain of the chest and back, fever, myalgia, arthralgia and rigors) and non-specific symptoms (rash, fatigue, dyspnea, throat tightness, dizziness/hypotension, vomiting, diarrhea) [35]. In mild cases, flu-like symptoms are present. In severe cases patients may develop aseptic meningitis, seizures, acute respiratory distress syndrome, renal failure, arrhythmia, cardiomyopathy, heart failure, hemophagocytic lymphohistiocytosis and macrophage activation syndrome [89]. CRSs appear on the first known administration and usually quickly disappear with repeated exposures [27]. They can be weakened or prevented by premedication with corticosteroids, acetaminophen and decelerating infusion [7]. CRS may be considered the most severe end of a spectrum including IRR. These reactions are more common after intake of rituximab, alemtuzumab, trastuzumab and cetuximab [34].

### 3.3. IgE-Mediated Reaction (Type I)

IgE-mediated reaction (type I) are characterized by quick onset of symptoms and signs (minutes to a few hours following intake) such as flushing, pruritus, rash, urticaria, throat tightness, shortness of breath, nausea, vomiting, cramping abdominal pain, diarrhea, hypotension and life-threatening anaphylaxis (cardiovascular collapse). When subcutaneous mAbs are given the reactions may occur several hours after exposure [90,91]. The clinical manifestations are caused by the release of mast cells/basophils mediators, including tryptase. Tryptase and skin test with a non-irritating concentration of mAb are indicators of this type of reaction [35]. IgE-mediated reactions do not appear at the first exposure, with the exception of cetuximab, in which pre-formed IgE antibodies due to a previous tick bite directed against an oligosaccharide, galactose-alpha-1,3-galactose (alpha-gal), present on the murine F(ab0)2 portion of cetuximab, could elicit a delayed HSR at first dose [92]. Skin tests and tryptase measurement shortly after the reaction could help to identify IgE-mediated reactions [93]. Note that mAbs may trigger mixed reactions characterized by elements of CRS (e.g., fever) as well as elements of an IgE-mediated reaction (high tryptase, positive skin testing).

### 3.4. IgG-Mediated Reactions

IgG-mediated reactions are still a matter of debate. Anti mAb IgG have been demonstrated for infliximab and adalimumab. The prevalence of antibodies against infliximab in children has been evaluated [94,95] and is around 35%.

The existence of IgG-mediated reactions has been hypothesized for mAbs such as infliximab, but not clearly demonstrated. The reaction occurs usually after several exposures and symptoms may be like those of IgE-mediated reactions as mast cells/basophils are activated. Skin test should be negative. These antibodies have been associated with reduced efficacy of infliximab through increased clearance or blocking antibody binding sites [35], as well as HSRs [96].

Serum sickness-like reaction (type III), the most frequent manifestation of a non-immediate HSR to mAbs, arises when immune complexes (IgG/IgM) deposit in tissues causing local or systemic injury. The most common clinical presentation is represented by classical triad of fever, arthralgia and rash. Myalgia, malaise, fatigue, edema, conjunctival hyperemia, and purpura have also been reported. Symptoms usually arise from 5 to 7 days (range 1–14 days) after infusion and most frequently after at least one exposure [35].

### 3.5. Delayed Type IV Reactions

According to the Gell and Coombs classification, maculopapular exanthema is a delayed (Type IVb) reaction that may occur with infliximab and abciximab. Atopic dermatitis has not been reported [97]. EM, SJS, TEN, AGEP, DRESS, are uncommon and typically develop from one day to several weeks following exposure to the mAb in question [35].

### 3.6. Local Reactions

Reactions at the injection site with itching, warmth, burning, stinging, pain, erythema, urticaria, and edema may develop. They may appear immediately, but usually develop in 24–48 h. Frequency depends on the drug (anakinra 71%, ustekinumab 2.4%) [36]. It is induced by drugs that trigger mast cell degranulation [98].

## 4. Allergy Work-Up

The allergy work-up in cases of reaction to mAbs starts with a detailed clinical history. It can help clinicians to speculate on the underlying mechanism of the reaction and to identify those patients that should be properly investigated. Several mAbs can cause reactions during the first exposure to the drug. When IRR or CRS are suspected, no allergy tests should be performed. Severe delayed reactions (i.e., SJS, DRESS, TEN) lead to mandatory change of treatment independently from allergy evaluation. When a type beta reaction, and, in particular, when an IgE-mediated mechanism is suspected, patients have to be investigated by skin testing. In the case of convincing clinical history and negative skin testing results, a graded challenge protocol can be used in order to reach a confident diagnosis and to better distinguish those patients that should be treated by desensitization from those that could safely receive regular drug infusion.

**Figure 1:**
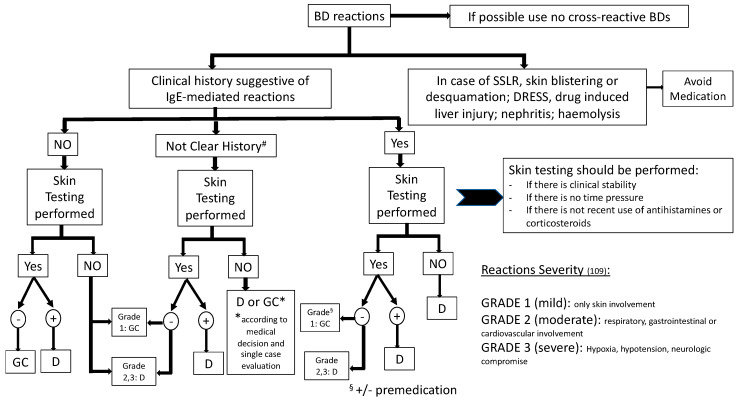
Allergy work-up. Adapted from [6]. GC = Graded challenge; RI = regular infusion; D = desensitization; SSLR = Serum Sickness Like reaction; DRESS = Drug reaction with eosinophilia and systemic symptoms. #In case of unclear history, always suspect an immediate reaction and perform a complete allergy work up.

However, because skin testing with mAbs has unknown sensitivity, if skin testing results are negative, the choice of desensitization is based on the severity of the initial reaction. Premedication followed by standard infusion can be provided when initial reaction was mild and skin testing was negative. Desensitization is recommended when the initial reaction was moderate to severe. Figure 1 summarizes a proposal for investigation and management in case of reactions to BDs [35,86,87,88,89,90,91,98,99,100,101,102,103,104,105,106,107,108,109].

### 4.1. Skin Testing

Ideally, skin testing should be performed 4–8 weeks after the reaction. Skin testing can have a negative result soon after an anaphylactic reaction because of depletion of vasoactive mediators. Over time, there is a progressive loss of specific IgE. Skin testing concentrations have not been standardized for most mAbs. We report the non-irritating concentrations of some BDs, used mainly in adult studies (Table 2). So far pediatric studies are limited in numbers.

For skin prick testing, a drop of the full-strength mAb is usually applied on the volar surface of the forearm. A positive result is defined by a wheal at least 3 mm in diameter larger than a negative control. Normal saline is use as negative control. Histamine (10 mg/mL) is used as positive control.

For IDT, non-irritating dilutions with sterile solution or normal saline are used. When the non-irritating concentration is not known 0.02–0.03 mL of a 1:1000 dilution followed by 1:100 and 1:10 dilutions are tested in sequence if the previous concentration result is negative. A positive result is obtained when the wheal, after 20 min, is at least 3 mm greater than the initial wheal.

Sometimes skin testing with some biologic drugs is not performed due to costs considerations. For practical and economic reasons, small aliquots of biosimilar should be prepared and used in the future [35,60,98].

### 4.2. In Vitro Tests

Serum specific IgE are not commercially available for mAbs. Recently, a positive correlation between positive IgE and IDT results has been described in adults who reacted to infliximab [100,101].

Basophil activation test (BAT) is measured by using mAbs to the specific activation markers CD63 and CD203c. So far BAT is used for research purpose only in case of immediate reactions [102,103]. In a pediatric study, infliximab hypersensitivity has been studied with BAT procedure at a drug concentration of 10 mg/mL with three different dilutions: 1:5; 1:125 and 1:125 [103]. In the acute phase, serum tryptase level are of paramount importance and very informative. Tryptase should be measured within 1-3 h from the reaction. Recently, the threshold of 11.4 μg/L has been abandoned and it is now recommended that an algorithm be used, suggesting a clinically relevant increase when tryptase at time of reaction is >2 + 1.2 × baseline tryptase [104]. Moreover, baseline tryptase should also be measured in order to rule out mastocytosis.

### 4.3. Drug Provocation Test

This test is recommended when there is a convincing or unclear history of mild cutaneous reaction, and a negative skin test. The drug provocation test (DPT) should be performed only when an alternative non-cross-reactive drug cannot be chosen. No definitive data on the frequency of cross-reactivity between two mAbs are available so far. Cross-reactivity may be due to structural similarity of two BDs (i.e., specific glycosylation of the Fc portion of IgG1 framework) and to target specificity (some antigen binding by different mAbs) [89]. The aim of the graded challenge is not to reach tolerance but to confirm or rule out hypersensitivity. In children, given the lack of ability to report subjective symptoms such as pruritus, nausea, dyspnea and/or throat or chest tightness, the initial dose administered is 1/100th of the therapeutic dose followed by 1/10 of the total dose and at last 9/10th. Each dose is administered at 30-min intervals [105]. The observation period is at least one hour after administration of the final dose. DPT should be performed by trained personnel to treat severe reactions and by expert allergists [110]. Up to 30% of DPT with BDs may have a negative DPT, therefore a DPT prior to desensitization may decrease the number of patients who need desensitization [106,107,108].

### 4.4. Desensitization

Rapid drug desensitization (RDD) induces a temporary tolerance to a drug which caused HSRs [111], that lasts only for the period (usually 24–48 h) that the drug persists in the patient’s system [112]. Therefore, patients need a new desensitization when the medication is not given daily [113,114].

It is well established that desensitization can be performed in patients with IgE-mediated drug allergy when an alternative, non-cross-reacting drug is not available [115,116]. Candidates for desensitization are: (1) all patients with positive skin tests to the mAb; (2) patients with unclear history and negative skin tests who had moderate (respiratory, gastrointestinal, cardiovascular involvement) or severe (hypoxia, hypotension, neurologic symptoms) reaction, or; (3) patients with convincing history who do not perform skin tests or with negative skin test results but reaction was moderate or severe.

The mechanisms of rapid IgE desensitization remain poorly understood, but there is evidence of generation of IgG-blocking antibodies, activation of inhibitory receptors, prevention of internalization of antigen/IgE/IgE receptor complex and polymerization of actin [117,118,119,120]. So, it seems that RDD can act by mechanisms that are similar to those of immunotherapy for aeroallergens [121]. RDD is contraindicated in type II and type III reactions, because there is a risk of activation and consumption of the complement system [112]. In severe type IV cell-mediated reactions, such as EM, AGEP, DRESS, TEN, or organ reactions, avoidance of the culprit drug is required since small amounts of the drug can provoke fatal reactions. Even if it is not indicated, RDD has been used to induce tolerance in subjects with non IgE-mediated reactions. It has been used for desensitizing patients with mild type IV delayed reactions [5,89]. Studies on phenytoin suggest that desensitization in cell mediated reactions can be mediated by T regulatory cells [122]. RDD is used for several categories of drugs including mAbs, that are often lifesaving or disease modifying, and leave allergic patients few alternative options [111,123]. Before starting RDD, skin testing with mAbs must be considered in order to improve understanding the exact mechanism and predict the risk of anaphylaxis. In malignant disease and autoimmune conditions, it is often urgent to reinstitute treatment with mAbs. Moreover, there is an increased likelihood of a false-negative reaction on skin testing, when performed shortly after HSR occurrence. Consequently, many authors do not consider that negative skin tests are a relative contraindication to perform desensitization [124,125]. RDD protocols consist of administration of increasing doses of the drug. The amount of the initial dose depends on the severity of the reported reaction. In children with anaphylaxis, the starting dose should be 1/1,000,000–1/10,000 dilution of the full dose [112]. Reactions during desensitization to mAbs occur in less than a third of procedures. Most reactions are mild, mainly cutaneous, and less severe than the initial reaction. The majority of reactions occur during the last steps [99,126]. Many protocols include premedication to reduce ADRs. Antihistamines and corticosteroids should not be administered to prevent IgE-mediated reactions since they mask initial symptoms and can delay treatment. In non-IgE-mediated reactions, antihistamines and corticosteroids can protect against mild-to-moderate HSRs during RDD [127]. Acetyl salicylic acid can be administered to patients who had flushing during the initial reaction. Montelukast is given to patients who suffer from bronchospasm during the reported reaction. Acetaminophen can be given to patients who experienced fever in the initial reaction [128]. The infusion must be halted at the onset of symptoms during desensitization and an appropriate treatment should be given [124]. After the acute reaction, has resolved, infusion is resumed from the step of the reaction. The protocol can be modified for future desensitization. A step should be added before the step of the breakthrough reaction. Moreover, additional medications can be given before the step at which the patient reacted [124,127]. Several RDD protocols to mAbs have been generated by different groups. The pivotal protocol is the one performed at Brigham and Women’s Hospital (Boston, MA, USA), considered the safest and most effective [35,93,111,126,129]. Castells et al. developed a standardized 12-step protocol for patients with HSRs to chemotherapeutic drugs, including a mAb (rituximab). All the patients underwent premedication. Three solutions (each 250 mL of water with 5% dextrose) were delivered in 12 consecutive steps at increasing infusion rates (Table 3). All patients received the target dose. Reactions mainly developed during the infusion of the solution 3, often during the last step. In cases of multiple desensitization procedures, most reactions were seen in the first two steps [129].

Many authors subsequently applied this protocol to several mAbs or proposed modified versions.

Patients who have experienced severe anaphylaxis during the treatment or symptoms early in the standard 12-step desensitization, underwent a 16-step protocol, which adds another bag containing 1/1000th of the full dose. The use of a 16-step (four bags) or a 20-step (five bags) protocol was reserved for high-risk patients [130]. Bavbek et al. [111] treated 17 patients who experienced HSRs to mAbs (14 to rituximab; 3 to cetuximab, infliximab, and trastuzumab, respectively) and noticed 13.5% adverse reactions, all of which were associated with rituximab and were less severe than the original reaction. RDD to mAbs (rituximab, trastuzumab, infliximab, cetuximab, bevacizumab, tocilizumab, ofatumumab, brentuximab, alemtuzumab) were successfully performed [99].

Brennan et al. used the standard 12-step protocol in 23 patients that had experienced HSRs to rituximab, infliximab, and trastuzumab. After a reaction during desensitization, patient-specific protocol modifications were performed before each subsequent desensitization [126]. Wong and Long analyzed the clinical reaction patterns of 25 patients with rituximab hypersensitivity. On the basis of clinical history, skin test reactivity and the patient’s previous desensitization outcomes, they performed drug desensitization using three continuous intravenous protocols that differed in starting dilution, steps [89,126], and duration (4.7–16 h). Nearly all patients with severe reactions to rituximab were successfully desensitized [64]. Furthermore, some desensitization protocols to subcutaneous mAbs, such as adalimumab and etanercept, were successfully performed [131,132,133]. RDD protocols to mAbs have been well defined in adults but there is limited experience in the pediatric population. Successful desensitization protocols in children has been reported for rituximab [125,134,135], infliximab [103,136] and tocilizumab [135]. Desensitization to infliximab had already successfully performed in 2001 for a 10-year-old boy with severe ulcerative colitis who had experienced an immediate severe anaphylactic/anaphylactoid reaction after repeated infusions of this drug. Two hundred and eight milligrams of drug (5 mg/kg) was administered, in 11 escalating increments every 15 min, ranging from 2 micrograms to 80 mg [136]. Aydogan et al. [135] reported a case of a 16-year-old boy with a steroid-resistant nephrotic syndrome who reacted to rituximab. Rituximab was administered using a 12-step RDD [123] with minor modifications. The desensitization procedure was performed thrice at one-week intervals without any reactions [135]. Justet et al. [137] described a 16-year-old girl with Still disease that was desensitized for tocilizumab. She experienced a grade 2 hypersensitivity, according to the World Allergy Organization grading system [21]. Tocilizumab (8 mg/kg: 480 mg) was given intravenously with a five-step protocol in 150 min. The first dose was 20 mg (4.1% of the total dose). There was no breakthrough symptom during the desensitization procedure. Caimmi et al. used a 13-step protocol to induce infliximab tolerance in a 14-year-old patient with severe ulcerative colitis who had experienced an anaphylactic reaction. The final cumulative dose was 251.11 mg, and the first one was 1/1,000,000 of the total dose. The dose was tripled at each step, every 15 min. RDD did not elicit any adverse reactions [103].

Dilley et al. [134] present three cases of pediatric patients. A 14-year-old boy with X-linked lymphoproliferative disease had a systemic reaction to rituximab for treatment of granulomatous lymphocytic interstitial lung disease. He underwent successful RDD to rituximab using a 12-step protocol described for the adult population. A seven-year-old boy with an orthotopic liver transplant developed a systemic reaction while receiving rituximab for treatment of post-transplant lymphoproliferative disease. Clinicians initially used a 16-step protocol. A breakthrough reaction occurred in the last step. Breakthrough reactions during the last step were also triggered by a modified 13-step protocol with slower infusion in the final step. Subsequently, they used a modified 12-step protocol with a final step not exceeding 2 mg/kg/h. A 23-month-old female developed urticaria while receiving rituximab for an opsoclonus myoclonus syndrome. The desensitization protocol was intended to reduce the step increase to no more than 0.5 mg/kg/h. The last step had a slower infusion of 2 mg/kg/h (Table 4). The procedure was well tolerated. It differs from the standard adult 12-step protocol mainly because of lower infusion rate of the last step.

Cansever et al. proposed a RDD protocol for two teenagers with high-grade B cell non-Hodgkin’s lymphoma who developed HSR to rituximab. This protocol involved 14-steps using three solutions with different concentrations. The initial dose was 1:50,000 of the total dose and the increment of the infusion rate was approximately 0.5 mg/kg/hour, with the final infusion rate not exceeding 2 mg/kg/hour [125], similar to the protocol proposed by Dilley et al. [134]. RDD is a costly and time-consuming procedure, possibly associated with serious reactions. This approach can be considered when valid alternatives are unavailable, because of lower efficacy or greater toxicity of alternative drugs, as well as when using mAbs. Weight-based RDD protocols with a slower final infusion rate than standard protocols are effective in reducing the risk of HSRs in children. Cohort studies on mAbs desensitization in children are lacking, and pediatric protocols simply adapt those used for adults [138]. The availability of standardized protocols is crucial for the success of this procedure.

## 5. Anti-Drug Antibodies (ADAs)

There is an increasing number of studies focused on new emerging approaches to predict, reduce or reverse biotherapeutic immunogenicity of BDs [139] while lowering the risk for hypersensitivity reactions. The development of ADAs may elicit hypersensitivity reactions by themselves or after the formation of a drug/ADA immune complex (IC). For example, in Type I hypersensitivity, IgE isotype ADAs are formed after a first or repeated exposure to the BD, leading to cross-linking of Fcε receptors on basophils, and mast cells provoking degranulation and release of mediators involved in anaphylaxis. This mechanism has been described in patients receiving infliximab [100]. Also, IgG isotype ADA can cause atypical anaphylaxis through activation and release of plated activating factor by neutrophils. Type III reactions occur subsequently to ADA/BD complex formation. IC deposition in blood vessels can cause thromboembolic phenomena as described with adalimumab [140]. Moreover, ADA production can neutralize the effects of BDs. Sometimes, ADA’s side effects can be reduced by using concomitant immunomodulatory agents. For example, patients with rheumatoid arthritis, spondylarthritis, and Crohn’s disease, and treated with anti-TNF-α agents, have benefited from concomitant methotrexate treatment [141].

## 6. Conclusions

mAbs are even more widely used in children in order to treat rheumatic, autoinflammatory and oncological diseases. True HSRs occur rarely, but when suspected a complete allergy work-up is mandatory. Besides the difficulties of performing a complete allergy work-up in children (i.e., poor tolerance of skin testing, risk for reactions during provocations, difficulty in reporting symptoms) as for any other drugs, BDs also have limitation due to costs of the medication itself. Today, pediatric studies, evaluating the diagnostic value of skin testing, and in-vitro testing in cases of suspected HSRs to BDs, are missing. In particular, sensitivity, specificity, positive and negative predictive values of those tests derived from adults need to be addressed in large samples of children.

## Figures and Tables

**Table 1:** Monoclonal antibodies approved by the Food and Drug Administration (FDA) and European Medicines Agency (EMA) for use in pediatric patients (www.ema.europa.eu; www.fda.gov).

Drug	FDA/EMA	Mechanism	Indications for Pediatric-Age Patients	Main Hypersensitivity Reactions
**Abatacept**	FDA >6 y/o EMA >6 y/o	T cells activation inhibition	EMA/FDA: Moderate-to-severe juvenile idiopathic arthritis (JIA) with inadequate response to other therapies including other anti TNF-alfa	No hypersensitivity reactions (HSR) or anaphylactic reactions in children [3] HSR in adults: 2.4 per 10,000,000 person-day [4]
**Adalimumab**	FDA/EMA from 2 y/o	Anti TNF-alfa	EMA: JIA, enthesitis associated arthritis, plaque psoriasis (PsO), Crohn’s disease (CD), non-infectious uveitis FDA: JIA, CD, non-infectious uveitis	HSR in 15% of children with JIA, 6.3% with psoriasis, 9.9% with CD [5] 7/10 children treated for JIA had antibodies against adalimumab correlated with a lower efficacy of therapy [6]
**Anakinra**	FDA/EMA >8 months >10 kg	Anti receptor IL-1	EMA: neonatal onset multisystem inflammatory disease/ chronic infantile neurological cutaneous and articular syndrome (NOMID/CINCA), mevalonate kinase deficiency (MKD), familial cold autoinflammatory syndrome (FCAS), sJIA FDA: NOMID/CINCA	Isolated cases of anaphylaxis [7,8]
Basiliximab	FDA 2-15 y/o EMA 1-17 y/o	Anti IL-2	EMA/FDA: Acute allograft rejection of kidney transplantation	
Benralizumab	FDA >12 y/o EMA >18 y/o	Anti IL-5R alpha	EMA/FDA: Eosinophilic severe asthma EMA considers safe for 12–18 y/o but no specific dose can be recommended	
Blinatumomab	FDA >0 y/o EMA >1 y/o	Anti CD3/CD19	EMA/FDA: B precursors acute lymphocytic leukemia, CD19 positive, Philadelphia chromosome negative, after allograft stem cells transplantation	
**Canakinumab**	FDA >4 y/o EMA >2 y/o	Anti IL-1	EMA: tumor necrosis factor receptor-associated periodic syndrome (TRAPs), Muckle-Wells syndrome (MWS), hyperimmunoglobumina D syndrome (HIDS)/MKD, NOMID/CINCA, FCAS, familial Mediterranean fever (FMF), sJIA FDA: TRAPs, HIDS/MKD, FCAS, MWS, FMF, sJIA	No HSR or anaphylactic reactions in children treated for FMF [9]
**Dupilumab**	FDA/EMA from 12 y/o	Anti IL-4/13	EMA/FDA: Eosinophilic severe asthma, severe atopic dermatitis, severe chronic rhino-sinusitis with nasal polyposis	No HSR or anaphylaxis reported, ongoing trial for age <12 y/o
Eculizumab	FDA >2 months EMA >5 kg	Anti C5	EMA: Postherpetic neuralgia (PHN), atipic hemolytic uremic syndrome (aHUS) FDA: aHUS; no safety and efficacy established in pediatric patients for PHN	
**Etanercept**	See indications	Anti receptor TNF-alfa	EMA: JIA with inadequate response to Methotrexate (MTX) >2 y/o; PsO >6 y/o; psoriatic arthritis (PA), enthesitis-associated arthritis >12 y/o FDA: moderate to severe JIA >2 y/o	Some reports of HSR to etanercept [10,11,12]
Golimumab	EMA >2 y/o e 10 kg FDA: adults >18 y/o	Anti TNF-alfa	JIA in association with MTX	
**Infliximab**	FDA/EMA >6 y/o	Anti TNF-alfa	EMA/FDA. Children: CD, ulcerative colitis (UC)EMA/FDA. Adults: rheumatoid arthritis (RA), ankylosing spondylitis, PA, PsO	Immediate HSR in 10% [13,14,15], delayed HSR less common [16]; well tolerated in children [17], 4.6% of IRR for rapid infusion in children [18]
Ipilimumab	FDA >12 y/o EMA >12 y/o	Anti CTLA-4	EMA: Metastatic melanoma (>12 y/o); renal carcinoma FDA: metastatic melanoma	
**Mepolizumab**	FDA >12 y/o EMA >6 y/o	Anti IL-5	EMA/FDA: Eosinophilic severe refractory asthma	Mild urticaria in 1/36 children [19], HSR in 3/621 but no anaphylaxis [20]
**Omalizumab**	FDA/EMA >6 y/o	Anti IgE	EMA/FDA: Moderate-to-severe persistent allergic asthma (>6 y/o), Chronic idiopathic urticaria (>12 y/o)	Anaphylaxis in <0.2% cases [21,22,23]. Potential risk factors: total doses, food allergy, female, urticaria
**Palivizumab**	See indications	Anti RSV	Newborn less 35 EG or 6 months of age or less at the beginning of season, with high risk of respiratory syncytial virus disease (bronchopulmonary dysplasia, congenital heart disease).	Generally well tolerated, a few and isolated HSR reported [24,25]
Ranibizumab	FDA/EMA Adults	Anti VEGF	Macular degeneration (adults) Preterm newborn for retinopathy of premature children: choroidal neo-vascularization	
**Rituximab**	See indications	Anti CD20	EMA/FDA: Adults: follicular and diffuse large B cells non-Hodgkin lymphoma, chronic lymphocytic leukemia; Wegener’s granulomatosis, severe RA, micropolyangitis, pemphigus vulgaris. FDA >2 y/o polyangitis; EMA >6 months for large B cell and Burkitt lymphoma *off label used in children for idiopathic thrombocytopenic purpura, steroid-dependent nephrotic syndrome steroid-dependent Schonlein–Henoch purpura	An infusion adverse event in 18/144 children, with 3 anaphylaxis (2%) [26], case-series with immediate HSR (mostly adults) [27,28,29]
**Tocilizumab**	FDA/EMA >2 y/o	Anti IL-6 receptor	EMA/FDA: polyarticular and systemic JIA, release cytokines syndrome from CAR-T therapy	Anaphylaxis developed in 3/128 children treated for rheumatic diseases [30] and in 4/1722 infusions for rheumatic diseases. A child with severe systemic JIA developed angioedema due to Ab to tocilizumab [31]
Ustekinumab	FDA/EMA Adults	Anti IL-12/IL-23	CD, UC, PsO >12 y/o	
Not approved for pediatric use but with on-going trial for compassionate use				
Bevacizumab	FDA/EMA adults	Anti VEGF	Colon rectal cancer, ovarian cancer Children: solid refractory tumors	
Brentuximab	FDA/EMA adults	Anti CD30	Hodgkin lymphoma, large cells lymphoma	
Cetuximax	FDA/EMA adults	Anti EGFR	Colon-rectal cancer Children: off-label central nervous system, tumors (glioma, astrocytoma)	
Gemtuzumab Ozogamicin	FDA/EMA >15 y/o	Anti CD33	Relapsed/refractory acute myeloid leukemia	
Natalizumab	FDA/EMA adults	Anti alpha-4 integrin	Multiple sclerosis	
Pembrolizumab	FDA/EMA adults	Anti PD1	Metastatic melanoma, non small cells lung carcinoma Children: metastatic melanoma, refractory solid tumor/lymphoma	
Trastuzumab	FDA/EMA adults	Anti HER-2	Osteosarcoma	
Vedolizumab	FDA/EMA Adults	Anti alpha-4/beta-7 integrin	CD, UC	
Reslizumab	FDA/EMA Adults	Anti IL-5	FDA/EMA: severe eosinophilic asthma FDA: eosinophilic granulomatosis with polyangiitis	

Approved and most-used mAbs in pediatric age patients is shown in bold. Abbreviations: atypical uremic hemolytic syndrome (aHUS), cryopyrin-associated periodic syndrome (CAPS), Crohn’s disease (CD), familial cold autoinflammatory syndrome (FCAS), familiar Mediterranean fever (FMF), hyper immunoglobumina D syndrome/mevalonate kinase deficiency (HIDS/MKD), juvenile idiopathic arthritis (JIA), microscopic polyangiitis (MPA), methotrexate (MTX), Muckle–Wells syndrome (MWS), neonatal onset multisystem inflammatory disease (NOMID)/chronic infantile neurological cutaneous and articular syndrome (CINCA), psoriatic arthritis (PA), paroxysmal nocturnal hemoglobinuria (PHN), plaque psoriasis (PsO), rheumatoid arthritis (RA), tumor necrosis factor receptor-associated periodic syndrome (TRAPS), ulcerative colitis (UC).

**Table 2:** Non-irritant skin testing concentrations with monoclonal antibodies. Adapted from [34].

Monoclonal Antibody	Skin Prick Test Concentration (mg/mL)	Intradermal Test Concentration (mg/mL)
Adalimumab	40	0.04–0.4–(4–40)
Anakinra	1502	15–150
Bevacizumab	5	2.5–25
Cetuximab	(2)-10	(0.2)–1–10
Entanercept	25-(50)	(0.5)–0.1–1–5
Infliximab	(5)-10	0.01–0.1–1–10
Omalizumab	125	0.00125
Pertuzumab	1.6	0.16
Rituximab	10	1–10
Tocilizumab	0.2-2-20 (4.8)	0.002–0.02–0.2–2–20
Transtuzumab	21	2.1

**Table 3:** Desensitization protocol for rituximab. Adapted from [129].

	Volume	Concentration	Amount of Drug in Each Solution (mg)			
Solution 1	250 mL	0.034 mg/mL	8.510			
Solution 2	250 mL	0.340 mg/mL	85.200			
Solution 3	250 mL	3.377 mg/mL	844.303			
Step no.	Solution no.	Rate (mL/h)	Time (min)	Volume infused per step (mL)	Administration dose (mg)	Cumulative dose (mg)
1	1	2.0	15	0.50	0.0170	0.0170
2	1	5.0	15	1.25	0.0426	0.0596
3	1	10.0	15	2.50	0.0851	0.1447
4	1	20.0	15	5.00	0.1702	0.3149
5	2	5.0	15	1.25	0.4255	0.7404
6	2	10.0	15	2.50	0.8510	1.5914
7	2	20.0	15	5.00	1.7020	3.2934
8	2	40.0	15	10.00	3.4040	6.6974
9	3	10.0	15	2.50	8.4430	15.1404
10	3	20.0	15	5.00	16.8861	32.0264
11	3	40.0	15	10.00	33.7721	65.7986
12	3	75.0	186	232.50	785.2014	851.0000

**Table 4:** Desensitization protocol for rituximab in pediatric patients. Adapted from [134].

	Volume (mL)	Drug Per Bag (mg)	Concentration (mg/mL)			
Solution 1	250	2.06	0.008			
Solution 2	250	20.6	0.082			
Solution 3	250	205.189	0.821			
Step no.	Solution no.	Rate (mL/h)	Rate (mg/Kg/h)	Time (min)	Dose per step (mg)	Cumulative dose
1	1	1	0.0006	15	0.0021	0.0021
2	1	2.5	0.002	15	0.0052	0.0073
3	1	5	0.003	15	0.0103	0.0176
4	1	10	0.006	15	0.0206	0.0382
5	2	2.5	0.02	15	0.0515	0.0897
6	2	5	0.03	15	0.103	0.1927
7	2	10	0.07	15	0.206	0.3987
8	2	20	0.1	15	0.412	0.8107
9	3	5	0.3	15	1.0259	1.8366
10	3	10	0.7	15	2.0519	3.8885
11	3	20	1.3	15	4.1038	7.9923
12	3	30	2	482.5	198.0078	206.0001
Therapeutic Dose 206 mg						

## List of Acronyms

acute generalized exanthematous pustulosis (AGEP), acute lymphocytic leukemia (ALL), adverse drug reactions (ADR), ankylosing spondylitis (AS), anti-drug antibodies (ADAs), atopic dermatitis (AD), basophil activation test (BAT), biologic drug (BD), bowel inflammatory diseases (BID), bronchopulmonary dysplasia (BPD), central nervous system (CNS), chronic infantile neurological cutaneous and articular syndrome (CINCA), chronic lymphocytic leukemia (CLL), congenital heart disease (CDH), Crohn’s disease (CD), cryopyrin-associated periodic syndrome (CAPS), cytokine release syndrome (CRS), cytotoxic T-lymphocyte antigen (CTLA), disease-modifying antirheumatic drugs (DMARDs), drug provocation test (DPT), drug reaction with eosinophilia and systemic symptoms (DRESS), erythema multiforme (EM), European Medicines Agency (EMA), familial cold autoinflammatory syndrome (FCAS), familial Mediterranean fever (FMF), Food and Drug Administration (FDA), hyperimmunoglobumina D syndrome (HIDS), hypersensitivity reaction (HSR), idiopathic thrombocytopenic purpura (ITP), immune complex (IC), infusion-related reaction (IRR), interleukin (IL), intradermal tests (IDT), juvenile idiopathic arthritis (JIA), methotrexate (MTX), mevalonate kinase deficiency (MKD), microscopic polyangiitis (MPA), monoclonal antibodies (mAbs), Muckle-Wells syndrome (MWS), neonatal onset multisystem inflammatory disease (NOMID), plaque psoriasis (PsO), psoriatic arthritis (PA), rapid drug desensitization (RDD), respiratory syncytial virus (RSV), rheumatoid arthritis (RA), serum sickness like reactions (SSLR), Steven-Johnson Syndrome (SJS), toxic epidermal necrolysis (TEN), tumor necrosis factor (TNF), tumor necrosis factor receptor-associated periodic syndrome (TRAPS), ulcerative colitis (UC), Wegener’s granulomatosis (WG), World Health Organization (WHO).
